# Plasma apixaban levels in Chinese patients with chronic kidney disease—Relationship with renal function and bleeding complications

**DOI:** 10.3389/fphar.2022.928401

**Published:** 2022-12-08

**Authors:** Chun-fung Sin, Ka-ping Wong, Tsz-fu Wong, Chung-wah Siu, Desmond Y. H. Yap

**Affiliations:** ^1^ Department of Pathology, Queen Mary Hospital, The University of Hong Kong, Pokfulam, Hong Kong SAR, China; ^2^ Division of Cardiology, Department of Medicine, Queen Mary Hospital, The University of Hong Kong, Pokfulam, Hong Kong SAR, China; ^3^ Division of Nephrology, Department of Medicine, Queen Mary Hospital, The University of Hong Kong, Pokfulam, Hong Kong SAR, China

**Keywords:** plasma level, renal function, bleeding, chronic kidney disease, apixaban

## Abstract

**Introduction:** Accumulation of apixaban in plasma is a major concern in patients with chronic kidney disease (CKD). Studies that investigated plasma apixaban level in CKD patients and its association with clinically significant events are scarce.

**Methods:** Patients with CKD Stage 1–4 who were taking apixaban, either 2.5 mg BD or 5 mg BD were recruited. The peak and trough plasma apixaban level were measured after 2 h and 12 h of last dose respectively. The results were correlated with renal function and clinical events during the period of follow-up from 1 January 2018 to 31 October 2021.

**Results:** 141 patients (CKD Stage 1, *n* = 12; Stage 2, *n* = 74; Stage 3, *n* = 48, stage 4, *n* = 7) were included for analysis. The plasma peak and trough apixaban were significantly higher in patients with CKD stage 3 when compared with those having CKD stage 2 and 1 (peak levels: 223.4 ± 107.8 ng/ml vs. 161.0 ± 55.2 ng/ml vs. 126.6 ± 30.2 ng/ml; trough levels: 118.3 ± 67.9 ng/ml vs. 81.2 ± 33.0 ng/ml vs. 51.9 ± 31.1 ng/ml, *p* < 0.05 or all) in patients taking 5 mg BD. Plasma trough apixaban level was negatively correlated with eGFR in patients taking 5 mg BD (*r* ^2^ = −0.174, *p* < 0.001) and 2.5 mg BD (*r* ^2^ = −0.215, *p* < 0.05). The plasma peak and trough apixaban level correlated with PT (*r* ^2^ = 0.065, *p* = 0.003 and *r* ^2^ = 0.096, *p* < 0.01 respectively). Multivariate analysis showed that plasma trough apixaban levels were associated with the risk of bleeding complications (Odd ratio: 1.011, 95% CI:1.002–1.021, *p* = 0.023).

**Conclusion:** The plasma apixaban level shows a trend of increase with worsening renal function, and an increase in the plasma apixaban level is suggestive of an increased risk of bleeding complications in patients with CKD. Further large-scale prospective studies are needed to evaluate relationship between plasma apixaban level and renal function as well as safety outcome in CKD patients. Moreover, the role of drug level monitoring should be prospectively evaluated for dosage optimization and the minimization of bleeding risks in CKD patients.

## Introduction

Anticoagulation constitutes an integral component in the management of patients with atrial fibrillation (AF) and venous thromboembolism. Direct oral anticoagulants (DOACs) have gained popularity over conventional warfarin since their approval because of their proven efficacy and safety, avoidance of regular blood monitoring and fewer interactions with other medications or food. Apixaban is a DOAC with direct anti-Xa activity. Previous studies have demonstrated that apixaban treatment is associated with better efficacy in stroke prevention and a lower risk of bleeding complications compared to warfarin in patients with AF (Granger et al., 2011).

While patients on apixaban exhibit the lowest rate of renal excretion (∼27%) among patients on DOACs, the efficacy and safety of apixaban in patients with renal impairment remain controversial. In this context, previous studies have shown that apixaban is associated with better efficacy in stroke prevention but a reduced risk of bleeding compared to warfarin in patients with stage 4 or 5 chronic kidney disease (CKD) (Pathak et al., 2015; Schafer et al., 2018; Siontis et al., 2018). In the ARISTOTLE study, patients with severe renal impairment (creatinine clearance [CrCl] <25 ml/min) were excluded (Granger et al., 2011; Hijazi et al., 2016). One previous pharmacokinetic study recorded a 36% increase in the plasma level of apixaban in patients with end-stage renal failure undergoing hemodialysis (HD) (Wang et al., 2016), and patients on HD receiving DOACs were at greater risk for both bleeding complications and thromboembolic events (Chan et al., 2016).

In general, DOACs including apixaban only require a fixed-dose regimen without the need for regular drug monitoring. Although routine monitoring of the plasma apixaban level is not recommended, its assessment may be indicated in some special clinical situations, such as the occurrence of bleeding complications, before an invasive procedure or in patients with renal impairment (Conway et al., 2017; Douxfils et al., 2021). Measuring the plasma apixaban level in patients with renal impairment is potentially useful because these patients experience progressive increases in bleeding risk as their kidney function declines (Becattini et al., 2018). However, real-world data on the effect of renal function and plasma apixaban level as well as its association with bleeding complications are lacking. The plasma level of apixaban can be measured *via* chromogenic anti-Xa assessment using commercially available assays with reasonable accuracy and precision even at low plasma concentrations (Lessire et al., 2020). It is also recognized that substantial differences exist in thromboembolic events and bleeding risks according to ethnicity. In this context, Chinese patients often have a lower risk of thromboembolic events but a greater risk of bleeding compared to Caucasians (Shen et al., 2007; Tsai et al., 2013; Wang et al., 2018). These variations are clinically pertinent because this discrepancy may affect the risk-benefit ratio of using DOACs and thus the dosage administered or even the target plasma DOAC levels required in Chinese patients with CKD.

Based on these knowledge gaps, this study aimed to investigate the relationship between plasma apixaban level, renal function and clinically significant events (i.e., thromboembolic or bleeding complications) in Chinese patients with CKD. Such data will help to optimize apixaban dosing and improve the drug’s efficacy and safety in patients with renal impairment.

## Patient and methods

### Patients and retrieval of clinical data

This 3-year prospective, observational cohort study was approved by the Institutional Review Board of the University of Hong Kong/Hospital Authority Hong Kong West Cluster (IRB HKU/HAHKWC) (reference number: UW 22-086), and all recruited subjects provided written informed consent. Patients who received a stable dose of apixaban for stroke prophylaxis of AF were recruited from the outpatient clinic of the Department of Medicine of Queen Mary Hospital in Hong Kong. Recruited subjects were prescribed either 2.5 mg twice daily (BID) or 5-mg BID of apixaban according to their age, renal function, and body weight as recommended by the package insert. Briefly, patients were prescribed 2.5 mg BID of apixaban if they fulfilled 2 of the following dose-reduction criteria: 1) age of >80 years, 2) serum creatinine concentration of >133 μmol/L, or 3) body weight of <60 kg. Patients with a CrCl of <30 ml/min were also prescribed a reduced dose of 2.5 mg BID. Patient demographics, history of medical comorbidities, liver and renal biochemistry values, and clotting profiles were collected at the time of study enrollment. During outpatient clinic follow-up, drug compliance was assessed by pill-counting, which was completed by clinic staff, and the information of drug compliance was documented in patients’ medical records. Patients were excluded from this study if they were non-Chinese in ethnic origin or showed non-compliance with apixaban treatment (defined by <90% compliance during the total study period). Patients were followed up with every 8–12 weeks, depending on their clinical condition. During each follow-up visit in the outpatient clinic, the following data were captured and documented in the medical records: dosages of apixaban, concomitant medications, serum creatinine, CrCl according to the Cockcroft-Gault equation, and clinically significant events (i.e., thromboembolic events or bleeding complications). The formula of Cockcroft-Gault equation for calculating CrCl is as follows: (140—age) × body weight/serum creatinine level × 72 (× 0.85 if female) (Michels et al., 2010). Data concerning clinically significant events were captured retrospectively if these events occurred before the current follow-up session in the outpatient clinic. Thromboembolic events were defined as any clinical signs and symptoms of transient ischemic attack or stroke, or by any radiological evidence of new-onset ischemic stroke or thromboembolism. The International Society on Thrombosis and Hemostasis (ISTH) definition was used to define major and minor bleeding, respectively. In brief, major bleeding episodes were defined by a hemoglobin concentration of <8 g/dl or a drop in the hemoglobin concentration of >2 g/dl from baseline, and any limb-threatening or life-threatening bleeding. All other bleeding episodes were defined as minor bleeding events (Kaatz et al., 2015). The investigators responsible for assessing clinically significant events were unaware of the patient’s plasma apixaban level. Clinical data were retrieved from the electronic health systems (ePR of the Hong Kong Hospital Authority) and further verified by reviewing patients’ medical records. The study period was from 1st January 2018 to 31st October 2021 and clinically significant events, including recurrent ischemic/thromboembolic events and bleeding complications were analyzed during this period.

### Blood sampling and processing

The plasma apixaban level was measured when patients had taken apixaban for at least 1 week (more than 5 half-lives) to ensure a stable plasma apixaban level was achieved. The peak and trough whole-blood samples for assessing the plasma apixaban level were collected at 2 and 12 h after apixaban administration, respectively. The procedure of blood sampling and the processing of blood samples from patients were performed according to a method described in the literature (Gosselin et al., 2018). Briefly, vacuum plastic tubes containing 3.2% trisodium citrate were used to collect blood samples for testing. After blood collection, samples were centrifuged at 3,750 rpm for 10 min. Platelet-poor plasma was isolated and stored at or below −70°C for future testing.

### Measurement of coagulation parameters and plasma apixaban level

Paired coagulation screening tests—namely, prothrombin time (PT) and activated partial thromboplastin time (APTT) assessments, were performed on all samples using Sysmex CS5100 analyzer (Siemens Healthineers, Erlangen, Germany). We used Thromorel S reagent and Actin FSL Activated PTT reagent (Siemens Healthineers) as PT reagent and APTT reagent, respectively. The procedure of plasma apixaban level measurement was performed according to a method described in the literature (Gouin-Thibault et al., 2014). Briefly, the plasma apixaban level was measured with the BIOPHEN DiXal kit (Hyphen BioMed, Neuville-sur-Oise, France) using the Sysmex CS5100 analyzer according to the manufacturer’s instructions. The principle of the test was to use a chromogenic anti-Xa assay specifically calibrated for apixaban, and the plasma apixaban level was expressed in units of ng/ml. A quality control measure was enacted before measuring the plasma apixaban level in patients’ samples by measuring the apixaban level in a quality control material provided by the manufacturer. The quality control of the assay was considered satisfactory if the value of the apixaban level obtained from the quality control material fell within the acceptable range provided by the manufacturer.

### Data analysis and statistical analysis

All continuous variables were presented as mean ± standard deviation (S.D.) or ranges as appropriate. One-way analysis of variance (ANOVA) (for parametric testing) or an independent-samples Kruskal–Wallis test (for non-parametric testing) was used to assess differences in continuous variables between groups. Frequencies and percentages were used to present categorical variables. The chi-squared test or Fisher’s exact test was used to analyzed categorical data where appropriate. The relationship between plasma apixaban level and the results of coagulation screening tests (PT and APTT) was analyzed by Pearson correlation. The relationship between plasma apixaban level and renal function was analyzed by linear regression. Logistic regression was used to analyze the relationship between clinically significant events and plasma apixaban level as well as other clinical parameters, including age, dosage, CrCl, history of diabetes mellitus (DM), hypertension (HT), body weight, concomitant intake of interacting drugs, and prior history of ischemic stroke or ischemic heart disease. Univariate regression was performed first to evaluate the relationship between clinically significant events and individual clinical parameters; this was followed by the analysis with multivariate regression.

The relationship between the plasma apixaban level and clinically significant bleeding events was defined as the primary outcome. In the ARISTOLE trial, the annual rate of any bleeding events was 20.47% among patients receiving apixaban (Goto et al., 2014). Based on this assumption, a sample size of 100 patients was required herein to achieve an 80% power to determine the relationship between the plasma apixaban level and any clinically significant bleeding events by univariate logistic regression at a 95% confidence interval (CI).

All statistical analyses were performed using the SPSS software program (version 27; IBM Corporation, Armonk, NY, United States), and *p*-value of less than 0.05 was considered to be statistically significant.

## Results

### Patient characteristics

One hundred forty-three Chinese patients participated in this study. Two patients were excluded from subsequent data analysis due to non-compliance (i.e., less than 90% compliance with apixaban treatment during the whole study period). Thus, a total of 141 patients (CKD stage 1, *n* = 12; stage 2, *n* = 74; stage 3, *n* = 48; and stage 4, *n* = 7) were included in the final data analysis (Table 1). Forty-three patients (30.5%) received 2.5 mg BID of apixaban and 98 patients (69.5%) received 5 mg BID of apixaban. All patients received apixaban at a dose prescribed according to the package insert. Patients receiving 2.5 mg BID of apixaban were older and had higher CHADS2 scores, lower CrCl and body weights, as well as a higher prevalence of DM and HT compared to those who received 5 mg BID (*p* < 0.05 for all). DM nephropathy and HT nephropathy were the most common underlying causes of CKD.

**TABLE 1:** Baseline characteristics of patients receiving apixaban 2.5 mg and 5 mg BID.

			Apixaban 2.5 mg (*n* = 43)	Apixaban 5 mg (*n* = 98)	*p*-value	Overall (*n* = 141)
Demographic information						
Sex (male) (%)			22 (51.2)	75 (76.5)		97 (68.8)
Age, years, mean +/- SD (range)			82.1 ± 4.9 (67-98)	69.9 ± 8.3 (47-86)	<0.001	73.6 ± 9.3 (47-98)
Age >80 years old (%)			31 (72.1)	11 (11.2)	<0.001	42 (29.8)
Body Weight (kg), median (range)			57.0 (35.9–90.4)	69.0 (40.0–102.6)	<0.001	65.0 (35.9–102.6)
CHADS2 (mean +/- SD)			2.65 (0.87)	1.61 (1.23)	<0.001	1.93 (1.23)
Stages of chronic kidney disease (%)		1	1 (2.3)	11 (11.2)		12 (8.5)
		2	16 (37.2)	58 (59.2)		74 (52.5)
		3	20 (46.5)	28 (28.6)		48 (34.0)
		4	6 (14.0)	1 (1.0)		7 (5.0)
Laboratory parameters						
creatinine clearance (ml/min), mean (range)			48.74 (21–82)	68.64 (38–99)	<0.001	62.57 (21–99)
Creatinine (umol/L), mean (range)			120.1 (58–239)	141.9 (48–155)	<0.001	135.2 (48–239)
Creatinine >133 (umol/L) (%)			13 (30.2)	4 (4.08)	<0.001	17 (12.1)
PT (s), mean (range)			13.43 (11.2–29.0)	12.2 (10.8–16.9)		13.24 (10.8–29.0)
APTT (s), mean (range)			33.32 (25.0–62.7)	33.99 (24.1–51.6)		33.79 (24.1–62.7)
Medical comorbidity (%)						
Diabetes mellitus (%)			25 (58.1)	28 (28.3)	0.001	53 (37.6)
Hypertension (%)			35 (81.4)	53 (54.1)	0.001	88 (62.4)
Hyperlipidemia (%)			10 (23.3)	19 (19.4)	0.656	29 (20.6)
Ischemic heart disease (%)			7 (16.3)	17 (17.3)	1.000	24 (17.0)
Old stroke/cerebrovascular accidents (%)			2 (4.7)	15 (15.3)	0.094	17 (12.1)
Past history of cancer			6 (14.0)	14 (14.3)	1.000	20 (14.2)
Causes of chronic kidney impairment						
Diabetic nephropathy (%)			22 (51.2)	24 (24.5)		46 (32.6)
Hypertensive nephropathy (%)			14 (32.6)	32 (32.7)		46 (32.6)
Obstructive nephropathy (%)			0 (0)	2 (2.0)		2 (1.4)
Ischemic nephropathy (%)			1 (2.3)	0 (0)		1 (0.7)
Surgery related e.g. nephrectomy (%)			1 (2.3)	0 (0)		1 (0.7)
Drugs Interacting with Apixaban						
Diltiazem			4 (9.3)	13 (13.3)		17 (12.1)
Dronedarone			1 (2.3)	3 (3.1)		4 (2.8)
Overall			5 (11.6)	16 (16.3)		21 (14.9)

Twenty-one patients (14.9%) were taking either cytochrome P450 3A4 (CYP450 3A4) inhibitors and/or P-glycoprotein (p-gp) inhibitors─namely diltiazem and dronedarone (Table 1).

### Relationship between plasma apixaban level and dosage

Patients taking 5 mg BID of apixaban showed a significantly higher mean peak plasma apixaban level compared to those receiving 2.5 mg BID (185.3 ± 88.3 ng/ml vs. 132.6 ± 48.7 ng/ml; *p* = 0.001). The mean trough plasma apixaban level in patients receiving 5 mg BID was also numerically higher than that in those taking 2.5 mg BID, albeit without reaching statistical significance (95.3 ± 56.1 ng/m vs. 77.9 ± 37.8 ng/ml; *p* = 0.061) (Table 2).

**TABLE 2:** Peak and trough apixaban level in patients receiving different dosage of apixaban.

	Apixaban 2.5 mg BID (*n* = 43)	Apixaban 5 mg BID (*n* = 98)	*p*-value	Overall (*n* = 141)
Peak Plasma Apixaban Level (ng/ml), median (5-95th percentile)	133.6 (64.5–215.0)	175.7 (67.6–359.1)	0.001	159.5 (67.7–349.5)
Trough Plasma Apixaban Level (ng/ml), median (5-95th percentile)	84.5 (7.9–127.8)	94.8 (14.4–199.9)	0.061	92.5 (12.4–181.1)

### Relationship between plasma apixaban level and renal function

**FIGURE 1:**
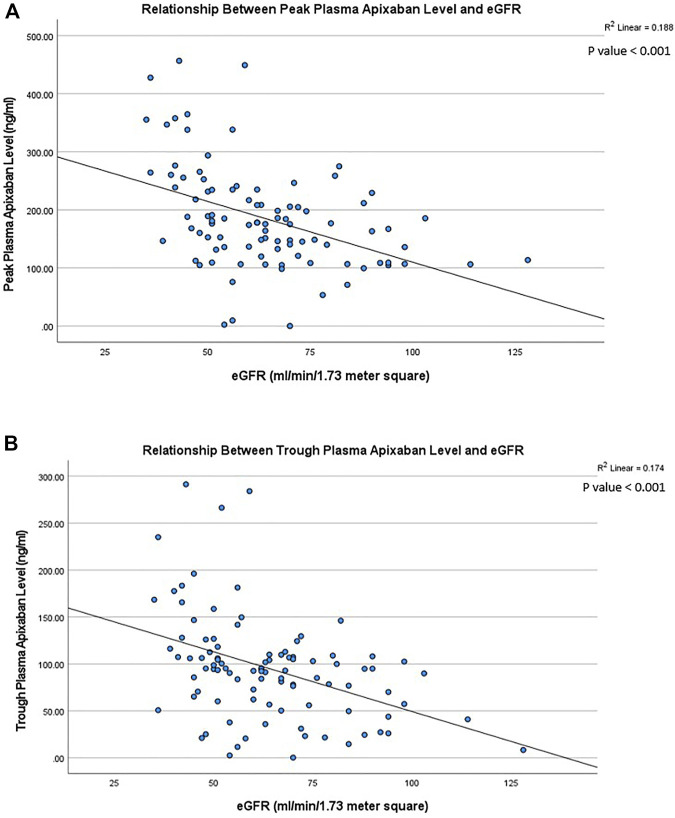
**(A)** Relationship between peak plasma apixaban level and creatinine clearance at 5 mg twice daily. The peak plasma apixaban level was negatively correlate with creatinine clearance (*r* ^2^: −0.188, *p* < 0.001) (*r* ^2^: coefficient of determination). **(B)** Relationship between trough plasma apixaban level and creatinine clearance at 5 mg twice daily. Linear regression analysis showed that the trough plasma apixaban level was negatively correlate with creatinine clearance (*r* ^2^: −0.174, *p* < 0.001) (*r* ^2^: coefficient of determination).

**FIGURE 2:**
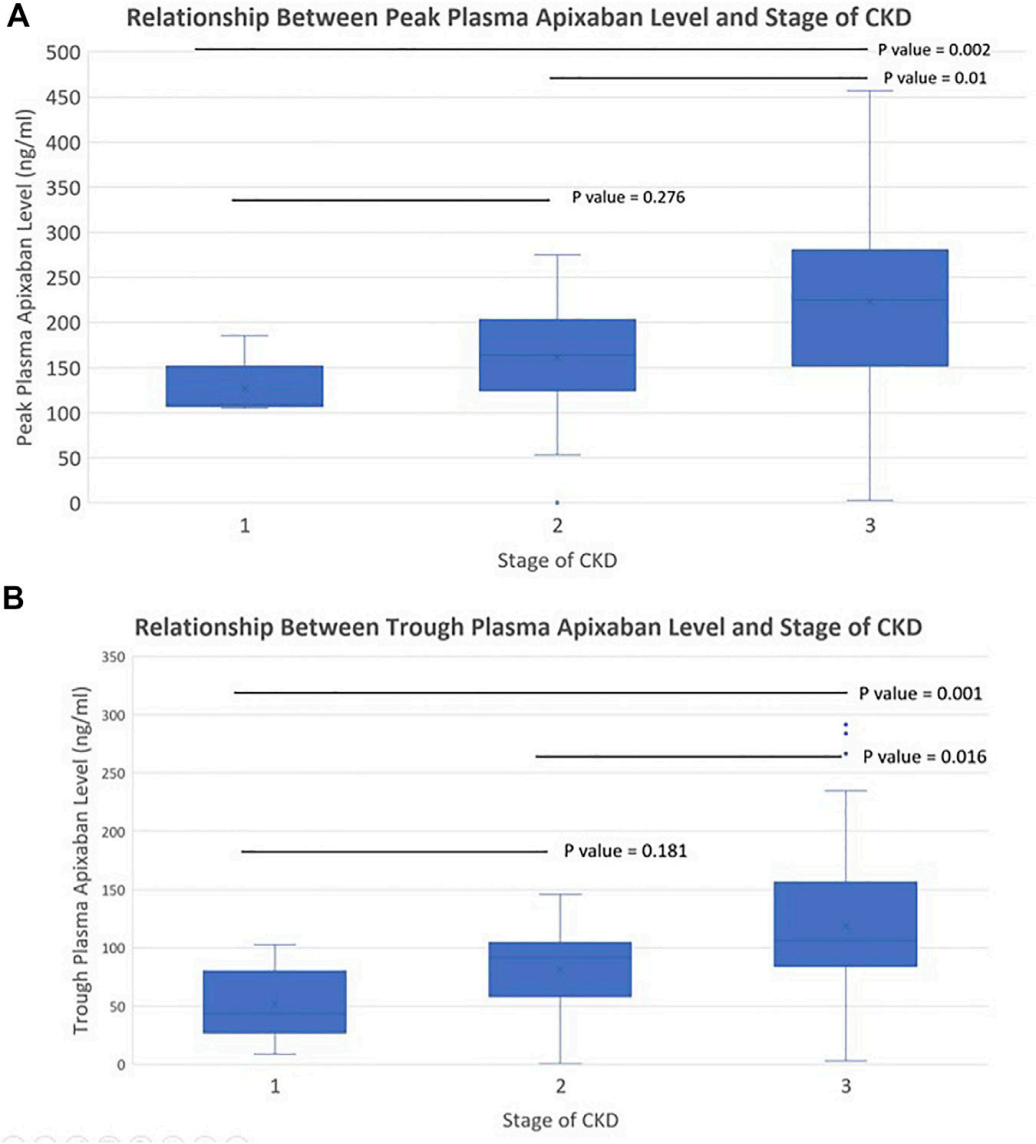
**(A)** Relationship between peak plasma apixaban level and stages of CKD (apixaban 5 mg twice daily). Difference of peak plasma apixaban level in patients with different stages of CKD. The peak level of stage 3 vs. stage 2 vs. stage 1 CKD patients was 223.4 ± 107.8 ng/ml vs. 161.0 ± 55.2 ng/ml vs. 126.6 ± 30.2 ng/ml, overall *p*-value <0.05; Difference between stage 3 CKD vs. stage 2 CKD, stage 3 CKD vs. stage 1 CKD, stage 2 CKD vs. stage 1 CKD: *p* = 0.01 vs. 0.002 vs. 0.276). **(B)** Relationship between trough plasma apixaban level and stages of CKD (apixaban 5 mg twice daily). Difference of trough plasma apixaban level in patients with different stages of CKD. The trough level of stage 3 vs. stage 2 vs. stage 1 CKD patients was 118.3 ± 67.9 ng/ml vs. 81.2 ± 33.0 ng/ml vs. 51.9 ± 31.1 ng/ml, overall *p*-value <0.05; Difference between stage 3 CKD vs. stage 2 CKD, stage 3 CKD vs. stage 1 CKD, stage 2 CKD vs. stage 1 CKD: *p* = 0.016 vs. 0.001 vs. 0.181).

In patients receiving 5 mg BID of apixaban, both the peak and trough plasma apixaban levels were negatively correlated with CrCl (*r* ^2^ = −0.188 and −0.174; *p* < 0.001 for both) (Figures 1A,B). Patients with stage 3 CKD had significantly higher mean peak and trough plasma apixaban levels compared to patients with stage 2 or 1 CKD (peak levels: 223.4 ± 107.8 ng/ml vs. 161.0 ± 55.2 ng/ml vs. 126.6 ± 30.2 ng/ml; trough levels: 118.3 ± 67.9 ng/ml vs. 81.2 ± 33.0 ng/ml vs. 51.9 ± 31.1 ng/ml; *p* < 0.05 for all) (Figures 2A,B). Analysis was performed again after excluding patients who were taking drugs known to interact with apixaban (CYP450 3A4 inhibitors and P-gp inhibitors); however, the peak and trough plasma apixaban levels remained negatively correlated with CrCl (*r* = −0.423 and −0.405; *p* < 0.01 for both) (Supplementary Figures S1A,B). Also, the peak and trough plasma apixaban levels were significantly higher in patients with stage 3 CKD than in those with stage 2 or stage 1 CKD (peak levels: 222.3 ± 112.4 ng/ml vs. 160.4 ± 57.0 ng/ml vs. 121.0 ± 23.0 ng/ml; trough levels: 118.3 ± 71.3 ng/ml vs. 82.9 ± 32.2 ng/ml vs. 50.0 ± 31.7 ng/ml; *p* < 0.05 or all) (Supplementary Figures S2A,B).

**FIGURE 3:**
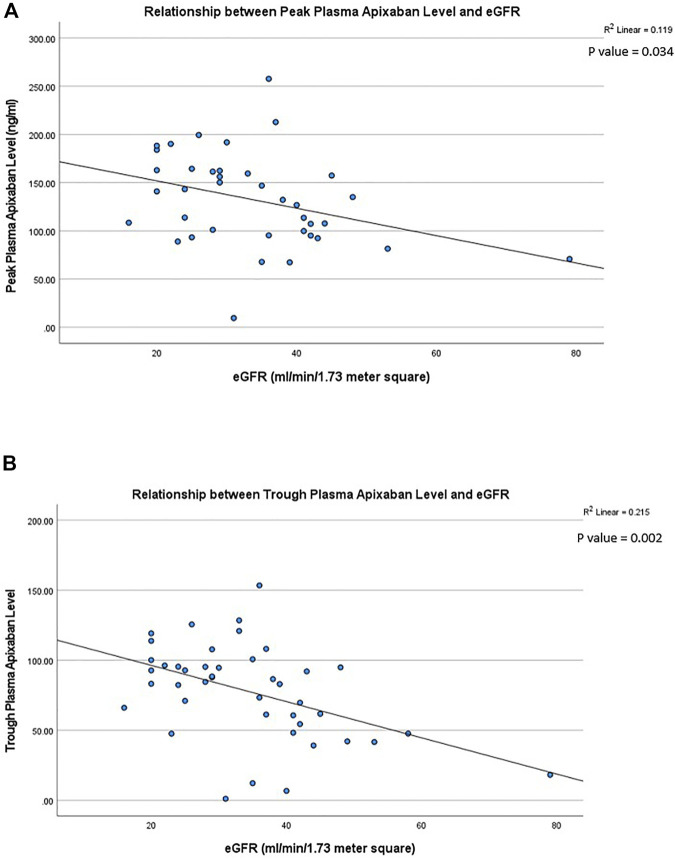
**(A)** Relationship between peak plasma apixaban level and creatinine clearance at 2.5 mg twice daily. The peak plasma apixaban level was negatively correlate with creatinine clearance (*r* ^2^: −0.119, *p* < 0.05) (*r* ^2^: coefficient of determination). **(B)** Relationship between trough plasma apixaban level and creatinine clearance at 2.5 mg twice daily. Linear regression analysis showed that the trough plasma apixaban level was negatively correlate with creatinine clearance (*r* ^2^: −0.215, *p* < 0.05) (*r* ^2^: coefficient of determination).

**FIGURE 4:**
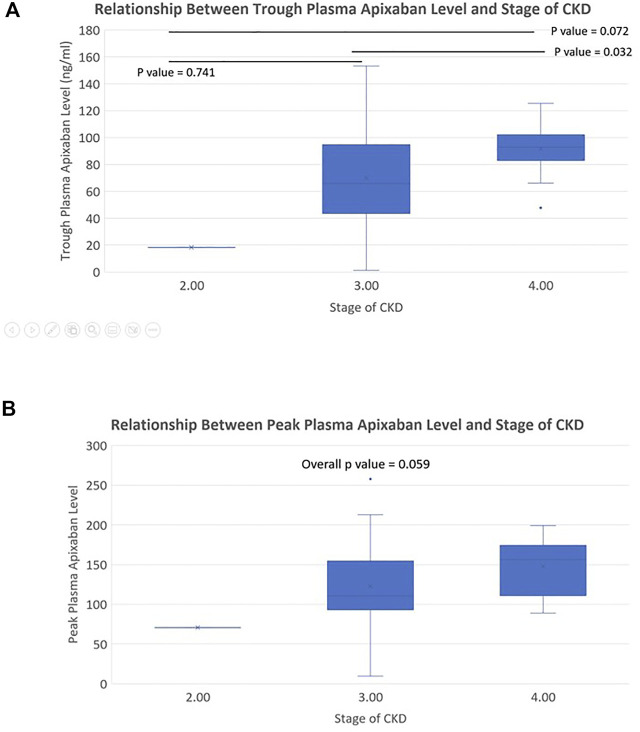
**(A)** Relationship between trough plasma apixaban level and stages of CKD (apixaban 2.5 mg twice daily). Difference of trough plasma apixaban level in patients with different stages of CKD. The trough level of stage 3 vs. stage 2 vs. stage 1 CKD patients was 91.6 ± 18.8 ng/ml vs. 70.1 ± 38.2 ng/ml vs. 18.2 ng/ml, overall *p*-value < 0.05; Difference between stage 3 CKD vs. stage 2 CKD, stage 3 CKD vs. stage 1 CKD, stage 2 CKD vs. stage 1 CKD: *p* = 0.032 vs. 0.072 vs. 0.741). **(B)** Relationship between peak plasma apixaban level and stages of CKD (apixaban 2.5 mg twice daily). Difference of peak plasma apixaban level in patients with different stages of CKD. The peak level of stage 3 vs. stage 2 vs. stage 1 CKD patients was 147.6 ± 35.1 ng/ml ng/ml vs. 122.9 ± 55.5 ng/ml vs. 70.5 ng/ml, overall *p*-value: 0.059.

In patients receiving 2.5 mg BID of apixaban, the peak and trough plasma apixaban levels were negatively correlated with CrCl (*r* ^2^ = −0.119 and −0.215, *p* < 0.05 for both) (Figures 3A,B). Patients with stage 4 CKD had significantly higher trough plasma apixaban levels compared to those with stage 3 or 2 CKD (Trough level: 91.6 ± 18.8 ng/ml vs. 70.1 ± 38.2 ng/ml vs. 18.2 ng/ml; *p* < 0.05). Patients with stage 4 CKD presented numerically higher mean peak plasma apixaban levels than those with stage 3 or 2 CKD, although this result did not reach statistical significance (peak level: 147.6 ± 35.1 ng/ml ng/ml vs. 122.9 ± 55.5 ng/ml vs. 70.5 ng/ml; *p* = 0.059) (Figures 4A,B). Analysis was performed after excluding patients who were taking drugs known to interact with apixaban (CYP450 3A4 inhibitors and p-gp inhibitors). The trough plasma apixaban level was negatively correlated with CrCl (*r* ^2^ = −0.176, *p* < 0.05), while the peak plasma apixaban level did not show a significant correlation with CrCl (*r* ^2^ = −0.075, *p* > 0.05) (Supplementary Figures S3A,B). The peak and trough plasma apixaban levels were numerically higher in patients with stage 4 CKD than in those with stage 3 or 2 CKD, although statistical significance was not reached (peak levels: 222.3 ± 112.4 ng/ml vs. 160.4 ± 57.0 ng/ml vs. 121.0 ± 23.0 ng/ml; trough levels: 87.6 ± 19.0 ng/ml vs. 70.0 ± 39.0 ng/ml vs. 18.2 ng/ml; *p* > 0.05 for all) (Supplementary Figures S4A,B).

### Relationship between plasma apixaban levels and coagulation screening tests

Both the peak and trough plasma apixaban levels were positively correlated with PT (*r* ^2^ = 0.065 and 0.096 for the peak and trough levels respectively; *p* = 0.003 and *p* < 0.01) (Supplementary Figures S5A,B). A positive correlation between APTT and the plasma trough apixaban level was also observed, while the same did not exist between APTT and the peak plasma apixaban level (*r* ^2^ = 0.041 and 0.005 for the trough and peak levels, respectively; *p* = 0.017 and 0.399) (Supplementary Figures S6A,B).

### Relationship between plasma apixaban levels and clinically significant events

The median follow-up time was 42 months (range, 2–45 months) and 7.8% of patients (11 out of 141 patients) had a follow-up period of less than 1 year. During the entire study follow-up period, a total of 6 episodes of recurrent ischemic/thromboembolic events, 24 episodes of minor bleeding, and 15 episodes of major bleeding were documented (Table 3; Supplementary Table S1). The annual event rates for recurrent ischemic/thromboembolic events and bleeding complications were 1.6% and 9.8% respectively. The median time from plasma apixaban level measurement to the occurrence of clinically significant events was 180 days. The subsequent analysis of bleeding events included both major and minor bleeding events.

**TABLE 3:** Clinically Significant Events in patients receiving apixaban 2.5 mg and 5 mg BD.

		Apixaban 2.5 mg BID	Apixaban 5 mg BID	Overall event rates for both 5 mg BID and 2.5 mg BID
Total	Stage 1 CKD	0 (0)	0 (0)	0 (0)
Recurrent ischemic events (%)	Stage 2 CKD	0 (0)	3 (5.2)	3 (4.1)
	Stage 3 CKD	2 (10.0)	1 (3.6)	3 (6.3)
	Stage 4 CKD	0 (0)	0 (0)	0 (0)
	Total events rate (all stages of CKD)	2 (4.7)	4 (4.1)	6 (4.3)
Bleeding complications (%)	Stage 1 CKD	0 (0)	4 (36.4)	4 (33.3)
	Stage 2 CKD	0 (0)	8 (13.8)	8 (10.8)
	Stage 3 CKD	6 (30.0)	17 (60.7)	23 (47.9)
	Stage 4 CKD	4 (66.7)	0 (0)	4 (57.1)
	Total events rate (all stages of CKD)	10 (23.3)	29 (29.6)	39 (27.7)

The mean trough plasma apixaban level was significantly higher in those patients who experienced bleeding complications compared to those who did not (112.0 ± 66.3 ng/ml vs. 81.5 ± 40.7 ng/ml; *p* = 0.032). The peak plasma apixaban level was also numerically higher in those patients who experienced bleeding complications compared to those who did not, although statistical significance was not reached (peak levels: 195.1 ± 105.8 ng/ml vs. 160.3 ± 69.1 ng/ml; *p* = 0.199) (Table 4).

**TABLE 4:** Mean peak and trough apixaban level in patients with or without bleeding events and recurrent ischemic/thromboembolic events.

Occurrence of events		Yes	No	*p*-Value
		Mean (± SD)		
Bleeding events	Peak apixaban level	195.1 ± 105.8	160.3 ± 69.1	0.199
	Trough apixaban level	112.0 ± 66.3	81.5 ± 40.7	0.032
Recurrent ischemic/thromboembolic events	Peak apixaban level	170.7 ± 54.6	171.1 ± 83.3	0.994
	Trough apixaban level	89.3 ± 36.3	90.2 ± 51.0	0.968

The peak and trough plasma apixaban levels showed a significant relationship with bleeding events [peak: odds ratio (OR) 1.005, 95% CI:1.000–1.010; *p* = 0.033 and trough: OR 1.012, 95% CI:1.004–1.020; *p* = 0.003] in the univariate regression analysis. Multivariate analysis further demonstrated a significant relationship between the trough plasma apixaban level and bleeding events after adjusting for age, body weight, CrCl, apixaban dosage, concomitant intake of interacting drugs, prior history of ischemic heart disease or ischemic stroke, history of DM, and hypertension (OR 1.011, 95% CI:1.002–1.021, *p* = 0.023). The peak and trough plasma apixaban levels did not show any significant difference among patients who experienced recurrent ischemic/thromboembolic events and those who did not (peak levels: 196.4 ± 105.6 ng/ml vs. 169.2 ± 83.7 ng/ml; trough levels: 93.2 ± 63.7 ng/ml vs. 89.8 ± 50.4 ng/ml; *p* > 0.05 for both) (Table 4). There was no significant relationship between peak and trough plasma apixaban levels and the rate of recurrent ischemia/thromboembolism (*p* > 0.05 for all).

## Discussion

Compared to other DOACs, renal excretion constitutes only 27% of apixaban elimination (Furie et al., 2012; Lu et al., 2014). Given such a pharmacological advantage, this drug is registered for stroke prophylaxis in AF and treatment for deep vein thrombosis with a CrCl as low as 25 ml/min (Furie et al., 2012; Lu et al., 2014). Thus, a substantial number of CKD patients who required DOAC treatment, especially those with relatively advanced renal failure, are prescribed apixaban. However, there is considerable variation in the peak and trough plasma concentrations, even in patients with normal renal function taking a stable dose of apixaban (Mavri et al., 2021). Hence, the use of apixaban remains an important clinical challenge in CKD patients, and the measurement of plasma apixaban level may serve as a useful way to optimize the dosage. In this study, nearly 40% of patients had CKD stage 3 or above. Our results suggested that both peak and trough plasma apixaban levels were negatively correlated with CrCl in patients receiving 5 mg BID of apixaban, and the trough level also increased with worsening CrCl in patients taking 2.5 mg BID of apixaban. Importantly, an increase in the trough plasma apixaban level was suggestive to be associated with a higher risk of bleeding complications in CKD patients.

The median peak and trough levels recorded from our cohort of patients were comparable to those listed on the package insert for both patients taking 2.5 mg BID and 5 mg BID (less than 10% of difference). However, the fifth to 95th percentile ranges of peak and trough plasma apixaban levels from our patients were different from those listed on the package insert (Alexander et al., 2016). The relatively small sample size of our study population could have contributed to the difference observed.

Dose adjustment based on drug level assay alone has not been established as a routine clinical practice for patients being treated with apixaban (Douxfils et al., 2021), and dosage modifications of apixaban are usually carried out according to age, renal function, and body weight (Granger et al., 2011; Lu et al., 2014). It is therefore a common practice to prescribe apixaban at lower dosages (i.e., 2.5 mg BID) in patients with more advanced CKD (Jain and Reilly, 2019). Indeed, patients receiving 2.5 mg BID of apixaban in this study had significantly lower CrCl and were older compared to those receiving 5 mg BID. Our present findings showed that patients receiving 5 mg BID of apixaban, irrespective of renal dysfunction, had significantly higher peak plasma levels than those taking 2.5 mg BID. Despite receiving the lower dose of 2.5 mg BID and having a lower trough plasma apixaban level compared to those taking 5 mg BID of apixaban, the trough plasma apixaban level even increased with worsening CrCl in patients taking a reduced dose of 2.5 mg BID. Such a result is likely related to the significant drug accumulation in patients with renal impairment, which affects the trough more than the peak drug concentration (Turpie et al., 2017). Data regarding the accumulation of apixaban in patients with advanced CKD have been conflicting. Wang *et al.* reported that there was a 36% greater apixaban exposure rate in patients with end-stage renal failure undergoing hemodialysis (Wang et al., 2016). However, another study on Asian patients found that there was no significant difference in plasma apixaban level between patients with a CrCl of >50 ml/min and those with a CrCl of <50 ml/min (Lin et al., 2020). One study from Japan reported no difference in the anti-Xa level between stage 3 and 4 CKD patients. (Tobe et al., 2020). However, the levels of anti-Xa activities were not compared between patients with stage 3 or 4 CKD and those with earlier stages of CKD (i.e., stages 1 and 2). Here, our data indicate that plasma apixaban level increases with the stage of CKD, despite the recommended dosage reduction. Importantly, our results suggested that the trough plasma apixaban level was associated with the risk of bleeding complications. Previous studies also found an increased major bleeding risk with deteriorating renal function over time for patients taking DOACs, though the number of patients taking apixaban was small in that study (Becattini et al., 2018). To further complicate the issue, CKD is also associated with high prevalence of HT and platelet dysfunction. Our finding is of particular concern in Chinese patients, who are recognized to have a higher propensity for bleeding compared to Caucasians (Shen et al., 2007; Tsai et al., 2013). The correlation between plasma apixaban level and bleeding complications is also in line with findings of studies on other ethnic groups (Testa et al., 2019; Skornova et al., 2021). However, one should note that the correlation between plasma apixaban level and CrCl was rather weak with small R square values obtained, which could be due to small sample size of this study. Nevertheless, further large-scale prospective studies are worthwhile to evaluate the relationship between plasma apixaban level and CrCl, as well as the role of measurement of plasma apixaban levels in minimizing hemorrhagic complications in CKD patients receiving apixaban, especially those from ethnic groups who have greater tendencies toward bleeding.

A previous study reported a poor relationship between plasma apixaban level and coagulation screening test results (Testa et al., 2016). However, our study found that both peak and trough plasma apixaban levels correlated with PT, though the correlation was rather weak with small R square values obtained. This could be explained by the fact that different PT reagents have different responses towards apixaban. One study documented a highly variable correlation coefficient between plasma apixaban level and PT among the different reagents tested (Gosselin et al., 2016). While our results showed that both peak and trough plasma apixaban levels correlated with PT, the alteration in PT was insensitive to the change in apixaban level (Samuelson et al., 2017). (Testa et al., 2016)Furthermore, there is a substantial difference in the sensitivity of various PT reagents toward apixaban, so the determination of apixaban exposure by PT/APTT or PT/APTT ratio is not practical (Dale et al., 2014; Bonar et al., 2016). Therefore, specific drug level assays that directly measure the apixaban concentration are clinically useful, especially in patients with deranged clotting profiles. The present data also showed that the trough plasma apixaban level but not the peak plasma apixaban level correlated with APTT, which corroborated with findings of other studies that reported a poor correlation between plasma apixaban level and APTT (Samuelson et al., 2017; Park et al., 2019).

The annual rate of recurrent ischemic/systemic embolic events was 1.6% in our patient cohort, which was comparable to that reported in the ARISTOTLE trial (Granger et al., 2011). Although a Caucasian study found that the trough plasma drug level of DOACs is associated with a greater preponderance of recurrent thrombotic events (Testa et al., 2018), our study did not find a significant relationship between the plasma apixaban level and rate of recurrent ischemic/thromboembolic events. The presence of risk factors other than a low plasma apixaban level (e.g., HT, DM or hyperlipidaemia) also contributes to the risk of recurrent ischemic/thromboembolism. Moreover, the small event rate of ischemic/systemic embolic events in our cohort may also result in a failure to detect any statistical significance.

There are several other limitations in our study. First, the sample size was small, with overall low event rates for ischemic/systemic embolic events and bleeding complications. The annual rate of major bleeding events was as low as 3.8% in this study. Thus, it was underpowered to assess the relationship between major bleeding events and trough plasma apixaban level. Second, this study only included patients who were ethnically Chinese, and patients with end-stage renal failure were not included. Moreover, the plasma apixaban level was assayed during the time of routine blood monitoring, but not at the time of clinically significant events. In addition, patients with stage 1–2 CKD composed a large proportion of the total patient population in this study (86 out of 141 patients, i.e., 61%) and 52% (74 out of 141) of patients having stage 2 CKD. The change in plasma apixaban level is much less dramatic in these patients when compared with those patients without CKD, so the results of this study would be potentially affected. The actual annual rates of recurrent ischemic/thromboembolic events and bleeding events were difficult to determine due to a wide range (2–45 months) of follow-up periods across our study population. Notwithstanding, our findings present real-world data on plasma apixaban exposure in patients with varying degrees of CKD and the relationship of such with clinically significant events during a follow-up period of approximately 3 years. The findings from our study provide important insight on monitoring the plasma apixaban level in patients with CKD to optimize the risk-benefit ratio when taking apixaban. Further large-scale, prospective clinical studies are needed to investigate the pharmacokinetic profile of apixaban and its relationship with clinical outcomes in CKD patients.

## Conclusion

The plasma apixaban level shows a trend of increase with worsening renal function, and an increase in the plasma apixaban level is suggestive of an increased risk of bleeding complications in patients with CKD. Further large-scale prospective studies are needed to evaluate relationship between plasma apixaban level and renal function, as well as safety outcome in CKD patients. Moreover, the role of drug level monitoring should be prospectively evaluated for dosage optimization and the minimization of bleeding risks in CKD patients.

## Data Availability

The raw data supporting the conclusion of this article will be made available by the authors, without undue reservation.
