# Supplementary material for: Plasma apixaban levels in Chinese patients with chronic kidney disease—Relationship with renal function and bleeding complications
Source: Front Pharmacol. 2022 Dec 8;13:928401. doi: 10.3389/fphar.2022.928401 (PMC9772439; doi:10.3389/fphar.2022.928401)

Supplementary tables and figures

Supplementary Table 1: Rate of major and minor bleeding in patients of different stages of CKD while taking apixaban 2.5 mg BD and 5 mg BD.

**Apixaban 2.5 (mg)**	**Stage 1**	**Stage 2**	**Stage 3**	**Stage 4**
Major bleed: n(%)	0 (0)	0 (0)	4 (20.0)	3 (50.0)
Minor bleed: n(%)	0 (0)	0 (0)	2 (10.0)	1 (16.7)
**Apixaban 5 (mg)**	**Stage 1**	**Stage 2**	**Stage 3**	**Stage 4**
Major bleed: n(%)	2 (18.2)	2 (3.4)	5 (17.9)	0 (0)
Minor bleed: n(%)	2 (18.2)	6 (10.3)	13 (46.4)	0 (0)

Supplementary Figure 1A: Analysis of was performed after excluding patients who taking interacting drugs. Relationship between peak plasma apixaban level and *creatinine clearance* at a dose of 5mg BID was analysis by linear regression. Result showed that the peak plasma apixaban level was negatively correlated with *creatinine clearance* (r^2: -0.179, p <0.001) (r^2: coefficient of determination).

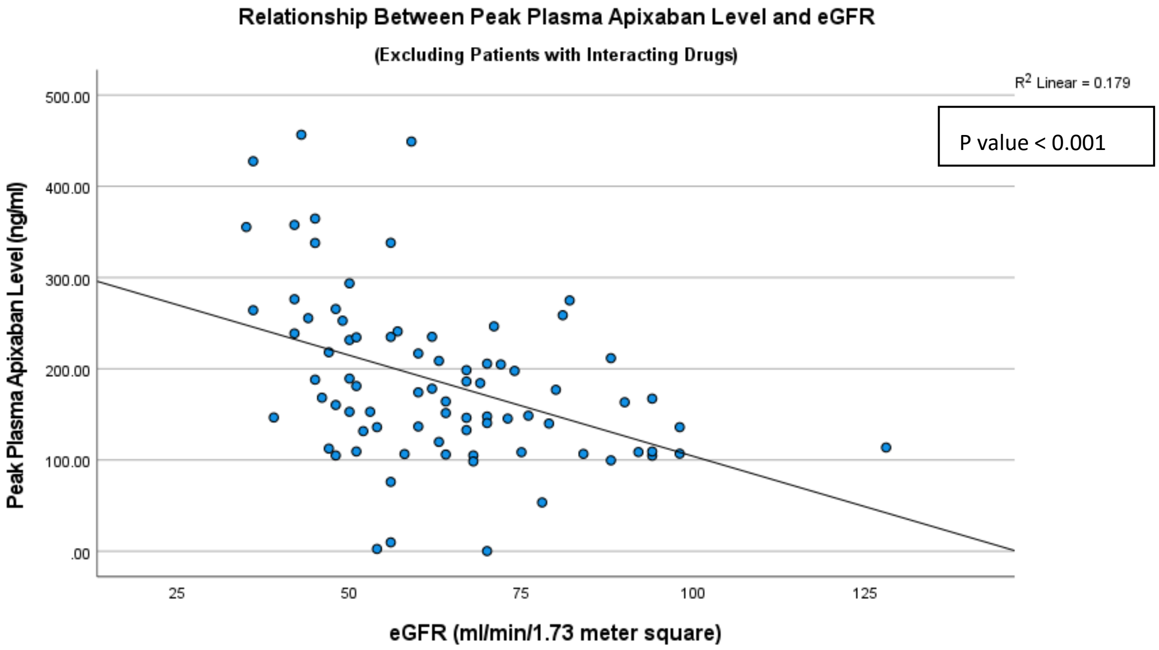

Supplementary Figure 1B: Analysis of was performed after excluding patients who taking interacting drugs. Relationship between trough plasma apixaban level and *creatinine clearance* at a dose of 5mg BID was analysis by linear regression. Result showed that the trough plasma apixaban level was negatively correlated with *creatinine clearance* (r^2: -0.164, p <0.001) (r^2: coefficient of determination).

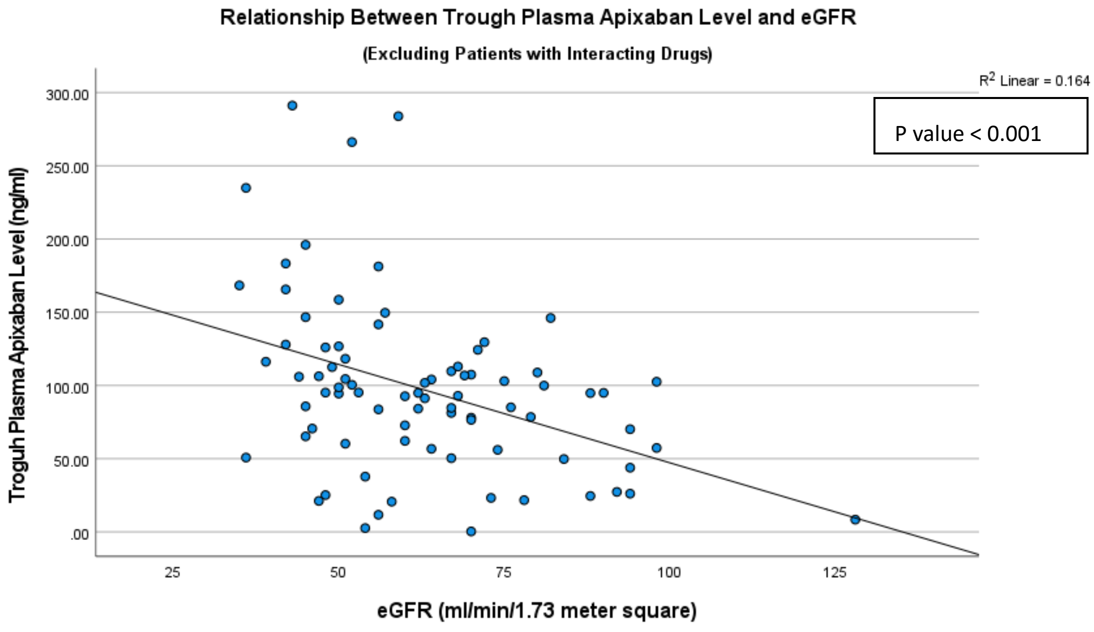

Supplementary Figure 2A: Analysis of was performed after excluding patients who taking interacting drugs. Difference of peak plasma apixaban level in patients with different stages of CKD at a dose of 5mg BID. The peak level of stage 3 vs stage 2 vs stage 1 CKD patients was 222.3±112.4 ng/ml vs. 160.4±57.0 ng/ml vs. 121.0±23.0 ng/ml, overall p value < 0.05; Difference between stage 3 CKD vs stage 2 CKD, stage 3 CKD vs stage 1 CKD, stage 2 CKD vs stage 1 CKD: p = 0013 vs 0.002 vs 0.102).

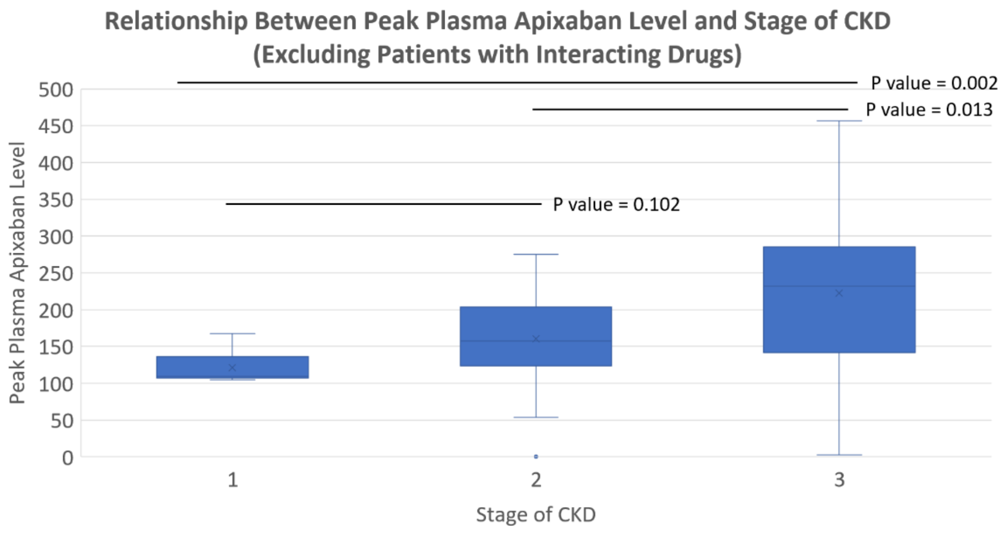

Supplementary Figure 2B: Analysis of was performed after excluding patients who taking interacting drugs. Difference of trough plasma apixaban level in patients with different stages of CKD at a dose of 5mg BID. The peak level of stage 3 vs stage 2 vs stage 1 CKD patients was 118.3±71.3 ng/ml vs. 82.9±32.2 ng/ml vs. 50.0±31.7 ng/ml, overall p value < 0.05; Difference between stage 3 CKD vs stage 2 CKD, stage 3 CKD vs stage 1 CKD, stage 2 CKD vs stage 1 CKD: p = 0018 vs 0.002 vs 0.077).

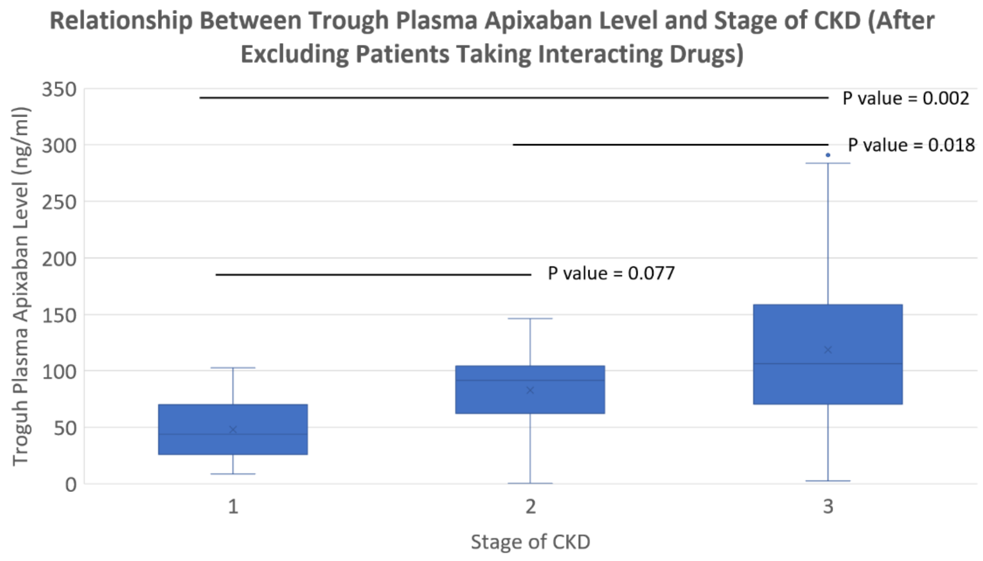

Supplementary Figure 3A: Analysis of was performed after excluding patients who taking interacting drugs. Relationship between trough plasma apixaban level and *creatinine clearance* at a dose of 2.5mg BID was analysis by linear regression. Result showed that the trough plasma apixaban level was negatively correlated with *creatinine clearance* (r^2: -0.176, p <0.05) (r^2: coefficient of determination).

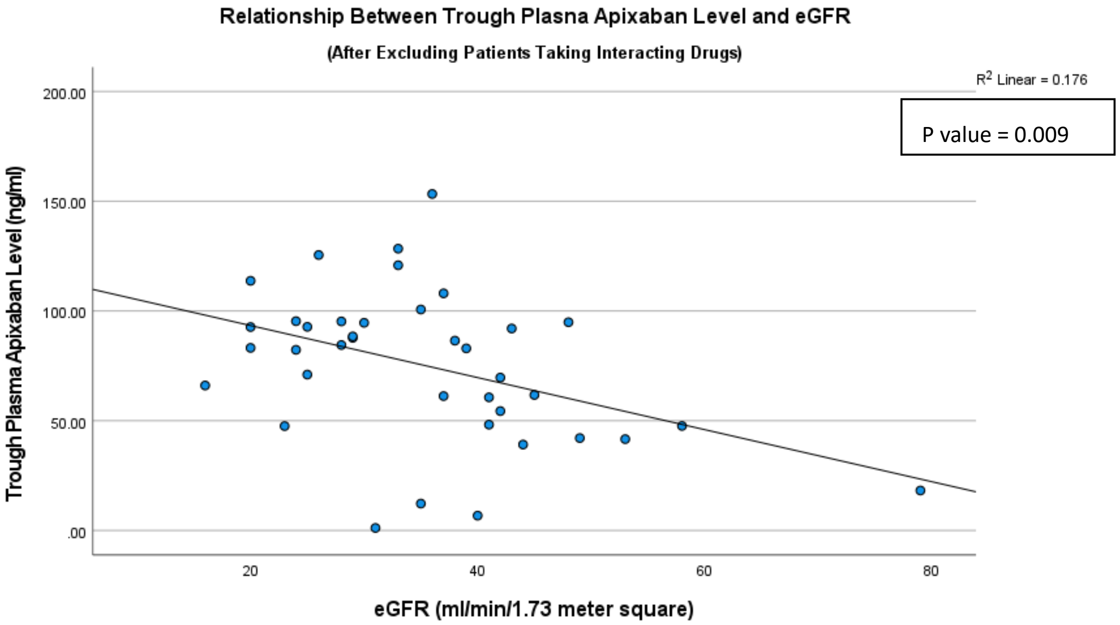

Supplementary Figure 3B: Analysis of was performed after excluding patients who taking interacting drugs. Relationship between peak plasma apixaban level and *creatinine clearance* at a dose of 2.5mg BID was analysis by linear regression. Result showed that there was no significant correlation between the peak plasma apixaban level and *creatinine clearance* (r^2: -0.075, p >0.05) (r^2: coefficient of determination).

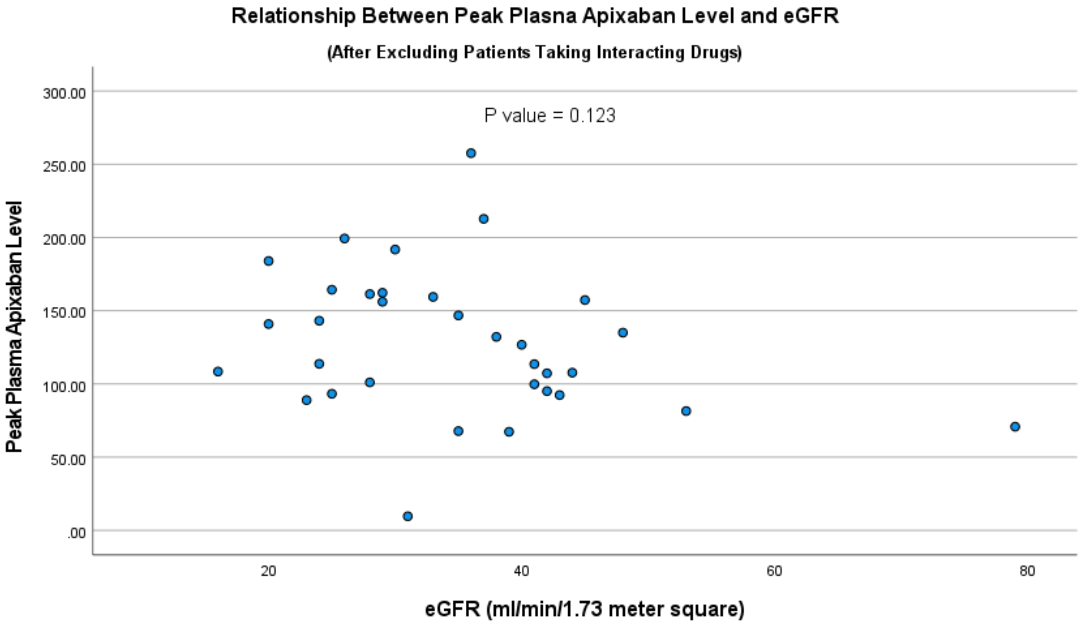

Supplementary Figure 4A: Analysis of was performed after excluding patients who taking interacting drugs. Difference of trough plasma apixaban level in patients with different stages of CKD at a dose of 2.5mg BID. The trough level of stage 3 vs stage 2 vs stage 1 CKD patients was 87.6±19.0 ng/ml vs. 70.0±39.0 ng/ml vs. 18.2 ng/ml, overall p value > 0.05).

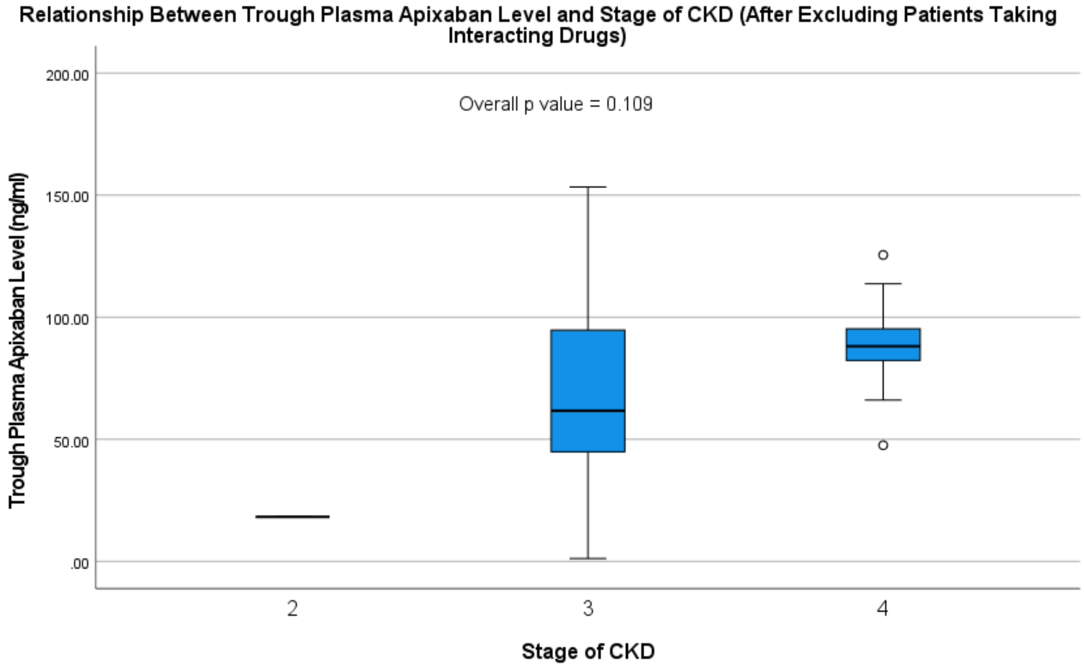

Supplementary Figure 4B: Analysis of was performed after excluding patients who taking interacting drugs. Difference of peak plasma apixaban level in patients with different stages of CKD at a dose of 2.5mg BID. The peak level of stage 3 vs stage 2 vs stage 1 CKD patients was 222.3±112.4 ng/ml vs. 160.4±57.0 ng/ml vs. 121.0±23.0 ng/ml, overall p value > 0.05).

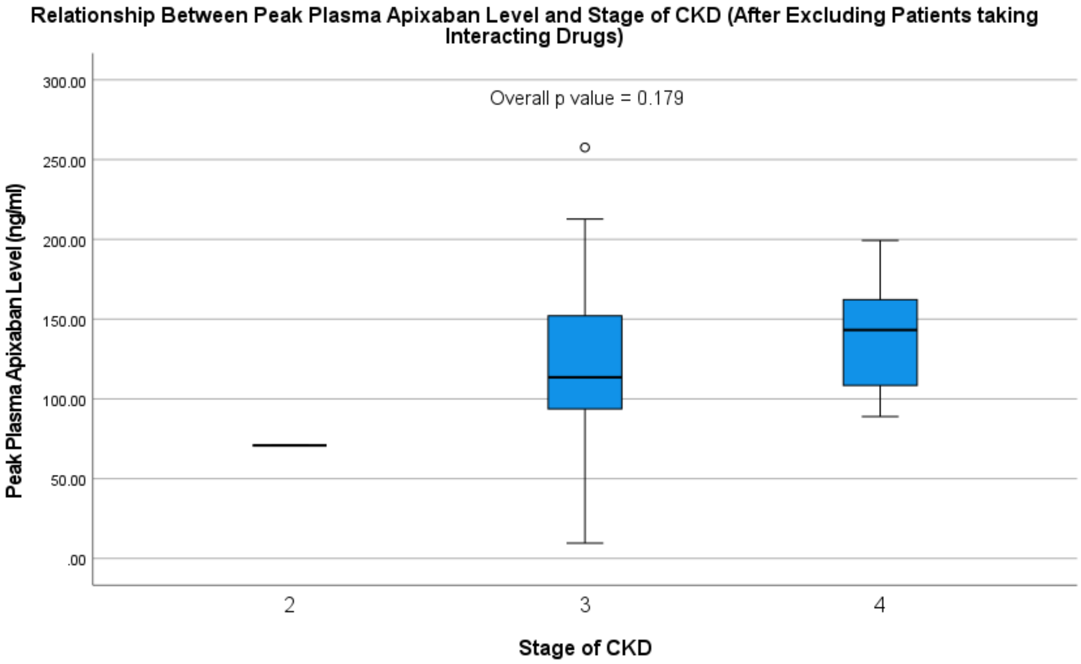

Supplementary Figure 5A: Relationship between PT and plasma peak apixaban level. Result showed that PT was positively correlated with peak plasma apixaban level (r^2: 0.065, p = 0.003; r^2: coefficient of determination).

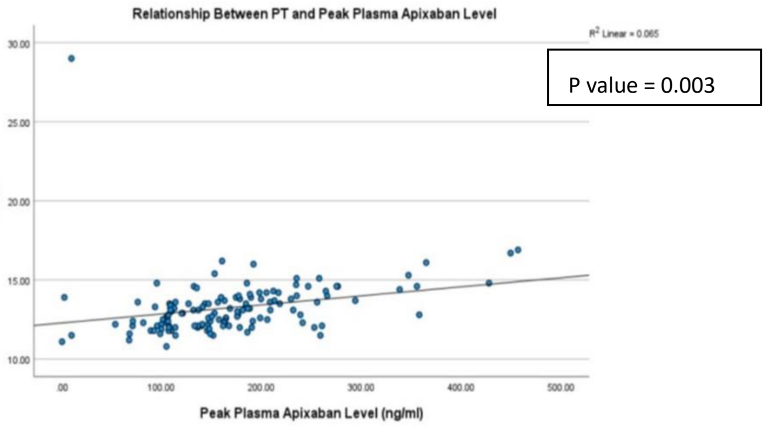

Supplementary Figure 5B: Relationship between PT and plasma trough apixaban level. Result showed that PT was positively correlated with trough plasma apixaban level (r^2: 0.096, p <0.001; r^2: coefficient of determination).

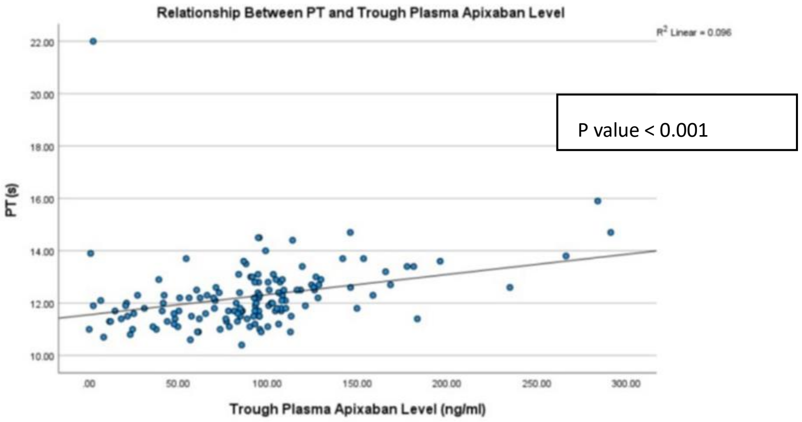

Supplementary Figure 6A: Relationship between APTT and trough plasma apixaban level. Result showed that APTT was positively correlated with trough plasma apixaban level (r^2: 0.041, p = 0.017; r^2: coefficient of determination).

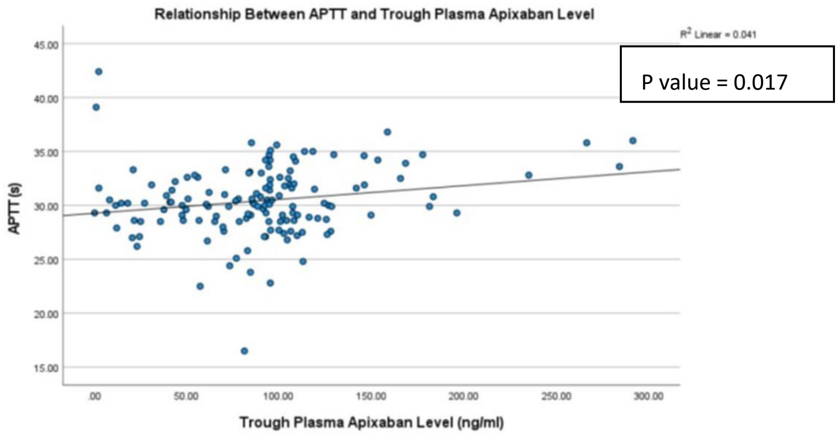

Supplementary Figure 6B: Relationship between APTT and peak plasma apixaban level. Result showed that APTT did not correlate with peak plasma apixaban level (r^2: 0.005, p:0.399; r^2: coefficient of determination).

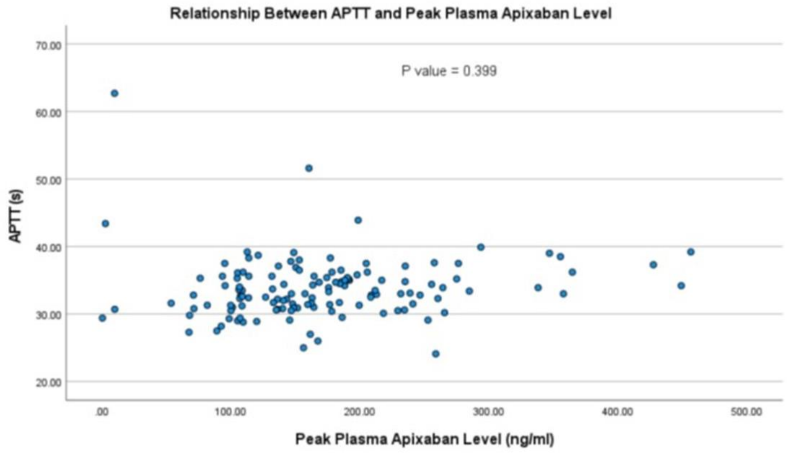

Supplement: Supplementary file 1 [file DataSheet1.pdf]
